# Neck mobility in the Jurassic plesiosaur *Cryptoclidus eurymerus*: finite element analysis as a new approach to understanding the cervical skeleton in fossil vertebrates

**DOI:** 10.7717/peerj.7658

**Published:** 2019-11-06

**Authors:** Tanja Wintrich, René Jonas, Hans-Joachim Wilke, Lars Schmitz, P. Martin Sander

**Affiliations:** 1 Section Paleontology, Institute of Geosciences, University of Bonn, Bonn, Germany; 2 Institute of Anatomy, University of Bonn, Bonn, Germany; 3 Institute of Orthopaedic Research and Biomechanics, Universität Ulm, Ulm, Germany; 4 Keck Science Department of the Claremont Colleges, Claremont, CA, USA; 5 Dinosaur Institute, Natural History Museum of Los Angeles County, Los Angeles, CA, USA

**Keywords:** *Cryptoclidus eurymerus*, Plesiosauria, Finite element model, Neck mobility, Range of motion, Marine reptile

## Abstract

The sauropterygian clade Plesiosauria arose in the Late Triassic and survived to the very end of the Cretaceous. Plesiosauria evolved the greatest species diversity of any marine reptile clade, attaining a global distribution. Plesiosauria consist of two clades, Rhomaleosauridae and Neoplesiosauria. Basal Neoplesiosauria have long necks with at least 30 cervicals, but show qualitative osteological evidence for a stiff neck. Here we quantify neck mobility in lateral, ventral, and dorsal directions based on finite element modeling of neck vertebrae from the Middle Jurassic plesiosaur *Cryptoclidus eurymerus*. We model the mobility in a single motion segment, consisting of two adjacent cervical vertebrae and the joints connecting them. Based on the model with a maximum intervertebral spacing of 3 mm, we find that in *Cryptoclidus*, the maximum angle of lateral deflection in the motion segment was 2°. The maximum angle of ventral deflection was 5° and of dorsal deflection was 5°. When these values are multiplied by the number of cervical vertebrae, it becomes apparent that neck mobility was limited in all directions. The maximum angle of total lateral deflection in the neck was 67°. The maximum angle of total ventral deflection was 148° and of total dorsal deflection was 157°. This raises the question of the function of such a long, multi-segment but immobile neck. We posit that the long neck served in hydrodynamic and visual camouflage, hiding the bulk of the body from the small but abundant prey, such as schooling fish and squid. Neck immobility may have been advantageous in withstanding strong hydrodynamic forces acting on the neck during predatory strikes.

## Introduction

Plesiosaurians are secondarily aquatic reptiles known from the Late Triassic to the Late Cretaceous (Storrs, 1993; Wintrich et al., 2017), which show a body design adapted extremely well to the aquatic environment. This is evidenced most clearly by the modification of the limbs into flippers used in a unique locomotion style, four-winged underwater flight. The clade Plesiosauria can be split into two subclades, the Rhomaleosauridae and the Neoplesiosauria, and the Neoplesiosauria can be further split into the Plesiosauroidea and the Pliosauridae (Benson, Evans & Druckenmiller, 2012; Benson & Druckenmiller, 2014). In general, there are three different body plans in plesiosaurs. First, there are forms with an extremely small head and a long neck, the plesiosauromorph body plan. Second, the pliosauromorph body plan is characterized by a massive and large head on a short neck. The third body plan, mainly seen in the Rhomaleosauridae, is a larger head and shorter neck than the small-headed and long-necked plesiosaurs, but a smaller head and a longer neck than the pliosaurs.

In the evolution of plesiosaurs, there is a remarkable change in the count of the cervical vertebrae (e.g., data compiled by Müller et al., 2010; Benson, Evans & Druckenmiller, 2012; Benson & Druckenmiller, 2014). More basal Plesiosauria, like *Rhaeticosaurus mertensi*, *Thalassiodracon hawkinsi*, *Eoplesiosaurus antiquior*, and *Avalonectes arturi* (Benson, Evans & Druckenmiller, 2012; Wintrich et al., 2017) have a relatively short neck with not more than 36 cervical vertebrae, whereas derived Plesiosauroidea of the subclade Elasmosauridae are famous for increasing the count of cervical vertebrae to over 70, like in *Aphrosaurus furlongi* that has up to 73 vertebrae (Zammit, Daniels & Kear, 2008). These are by far the highest number of cervical vertebrae of any vertebrate (Müller et al., 2010; Wintrich et al., 2017). Naturally, the function of such a long neck has received extensive discussion ever since the first complete plesiosaur skeletons became known to science nearly 200 years ago (Conybeare, 1824), as reviewed by Noè, Taylor & Gómez-Pérez (2017) and Troelsen et al. (2019). One thought is that the plesiosaur neck is immobile (Watson, 1924; Watson, 1951; Cruickshank & Fordyce, 2002) and was used for ambush hunting (Taylor, 1981; Massare, 1988; Massare, 1994). Furthermore, and contrary to Taylor (1981), Massare (1988) and Massare (1994), several authors (Taylor, 1987; McHenry, Cook & Wroe, 2005; Noè, Taylor & Gómez-Pérez, 2017) suggested a grazing-style feeding pattern on the sea bottom in Plesiosauria, especially in Elasmosauridae, requiring a mobile neck (Andrews, 1910; Brown, 1981). Besides these speculations, Taylor (1981) and Storrs (1993) proposed an upward S-shape curving neck posture for hunting prey from above the water surface while the plesiosaur’s body was submerged. This was taken further by Wilkinson & Ruxton (2011) who suggested that plesiosaurs had a hunting style similar to long-necked birds such as cormorants, relying on fast acceleration of the head on a highly mobile neck.

The question of the mobility of the neck is extensively discussed from a biomechanical perspective in the literature on mammals and dinosaurs, including birds (e.g.,  Putz, 1992; Boszczyk, Boszczyk & Putz, 2001; Buchholtz & Schur, 2004; Dzemski & Christian, 2007; Zammit, Daniels & Kear, 2008; Christian & Dzemski, 2011; Wilkinson & Ruxton, 2011; Cobley, Rayfield & Barrett, 2013; Stevens, 2013; Taylor & Wedel, 2013; Krings et al., 2014; Viglino et al., 2014; Molnar, Pierce & Hutchinson, 2014) but in a very limited way for plesiosaurs.

Vertebral centrum shape, neural arch morphology, especially of the zygapophyses, the space between two centra, the muscles, ligaments, and cartilages all impose limitations on the mobility of the vertebral column (Buchholtz & Schur, 2004; Niemeyer, Wilke & Schmidt, 2012; Stevens, 2013). Functional anatomy allows testing of hypotheses of neck mobility and limitations of neck range of motion (ROM) in plesiosaurs based on the cervical vertebral column and the morphology of the individual cervical vertebrae. Although a previous detailed study (Zammit, Daniels & Kear, 2008) had noted limited mobility of the plesiosaur neck based on functional morphology, this was done only in two dimensions using an analog model. In a more traditional approach, Noè, Taylor & Gómez-Pérez (2017) reviewed the osteology of the neck of long-necked plesiosaurians and concluded that the neck was most mobile in a ventral direction. This view is inconsistent with a recent case study focussing on the leptoclidid plesiosaur *Nichollssaura* which reconstructed a rather mobile and predominantly laterally flexible neck consisting of only 24 vertebrae based on the digital manipulation of 3D surface models of individual cervical vertebrae (Nagesan, Henderson & Anderson, 2018).

The current work expands on these studies by employing an approach from computational biomechanics, sourced from the field of human biomechanics (Yoganandan et al., 1996; Niemeyer, Wilke & Schmidt, 2012). Specifically, we use three-dimensional finite-element models developed for investigations of human spine function that include soft part anatomy to model intersegmental mobility. This approach goes beyond osteological arguments (Noè, Taylor & Gómez-Pérez, 2017), the manipulation of analog models (Zammit, Daniels & Kear, 2008), and of digital osteological models (Nagesan, Henderson & Anderson, 2018) of the kind first successfully employed in the study of dinosaur neck biomechanics (e.g., Stevens, 2013). Our model organism is the cryptoclidid plesiosaur *Cryptoclidus eurymerus* from the Middle Jurassic of England (Andrews, 1910) which is well represented by mounted skeletons in museum collections such in the Goldfuß Museum of the Institute of Geosciences, University of Bonn, Bonn, Germany (collections acronym IPB).

For a better understanding of the morphological, mechanical, and biological characters to be discussed in our paper, we first review the most important definitions in functional morphology of the vertebral column in general and the neck in particular. We then discuss previous thoughts and our assumptions about the nature of the connection between the vertebral centra, specifically the type of joint. We conclude that in plesiosaurs, the centra were closely spaced at only a few mm apart and connected by an intervertebral disc (IVD) which we then implement in our finite element models.

The aim of our study is to estimate the ROM in the cervical vertebral column of an extinct marine reptile, the plesiosaur *Cryptoclidus eurymerus*, using finite element modeling. Comparison of the material settings is used here to evaluate the ROM and therefore discuss the relative mobility and functional implications of the cervical vertebral column which gives insights into the paleobiology of plesiosaurs.

## Terminology and anatomy

### Degrees of freedom

In a three-dimensional space, six degrees of freedom are possible, three involving rotation and three involving translation. However, translation can be neglected in the function of the vertebral column along its axis. Therefore, only three degrees of freedom affect the vertebral column. These degrees are X_r_, Y_r_, and Z_r_ for a centrum with intervertebral discs (as in plesiosaurs, see below). Whereas X_r_ describes dorsoventral movement around a mediolaterally oriented axis, Y_r_ describes the rotation or twist of a segment around a horizontal axis, and Z_r_ describes the lateral movement of a segment by rotation around a vertical axis. Regarding these degrees of freedom, actual movement is limited by different osteological structures. X_r_ is affected by the centrum morphology, the distance between the centra, the zygapophyses, the neural spine, and the ribs. Y_r_ is affected only by the zygapophyses, and Z_r_ is affected by the distance between the centra, the zygapophyses, and the ribs.

### The motion segment

Two adjacent vertebrae that are connected by muscles and ligaments, and their articulations are variously called a ’motion segment’ or a ’Junghans functional unit’. Here we use the term ’motion segment’. The motion segment is the smallest definable biomechanical unit in the vertebral column. Here, we first determine the angle of maximum mobility of the motion segment for *Cryptoclidus* and then derive the mobility in the entire neck.

### Joints in the amniote vertebral column

In order to determine the true mobility of the plesiosaur neck, we need to discuss anatomical features in the vertebral column. In particular, the question of how two vertebrae are connected must be considered because the joints between contiguous vertebrae have an important influence on function and therefore on mobility (Currey, 2002).

The tetrapod joint is a mobile connection between two or more bony or cartilaginous skeletal elements. Usually, joints can be divided into two main groups, diarthroses on the one hand, and amphiarthroses and synarthorses on the other. Diarthroses are ‘real joints’ and are also called synovial joints. Diarthroses are a freely movable system. In the vertebrae, for example, the zygapophyses are synovial plane joints. Amphiarthroses and synarthroses are connections between bones that are made of fibrous connective tissue or cartilage, allowing limited ROMs. Examples of synarthroses are sutures between bone and examples of amphiarthroses are intervertebral disks (IVDs). Among extant amniotes, lepidosauromorph reptiles and crocodiles have synovial joints connecting the vertebral centra. In the basal lepidosaur *Sphenodon*, there is a continuous notochord (Wettstein, 1962), whereas birds have a unique type of joint with a saddle-shaped surface and no nucleus pulposus. Finally, in mammals, there is an IVD in this position. The mammalian IVD consists of an *annulus fibrosus* and a *nucleus pulposus*. The nucleus pulposus is a round structure consisting of loose fibers suspended in a mucoprotein gel, whereas the annulus fibrosus consists of fibrocartilage.

### Centrum and intervertebral joint morphologies and tissues

Among amniotes, different shapes of the centrum evolved, some of which are convergent (Romer, 1956). The plesiomorphic condition is the *amphicoelous* centrum with deeply concave, funnel-shaped anterior and posterior faces, sometimes connected by a small foramen for the notochord. If the centrum is anteriorly and posteriorly flat, it is called *platycoelous* (synonym *acoelous*), whereas a centrum with an anteriorly concave and posteriorly convex face is called *procoelous*; the other way around it is called *opisthocoelous*. These different centrum shapes influence the mobility and also limitation of movement of the vertebral column. Plesiosaur centra are typically reported to be platycoelous or weakly amphicoelous, as in the cervicals of *Cryptoclidus* (Benson, Evans & Druckenmiller, 2012; Benson & Druckenmiller, 2014).

Furthermore, for understanding vertebral column mobility and neck mobility in amniotes, the distance between the centra and the question of the presence of different cartilage tissues and intervertebral discs are important. Due to the fact that soft tissue is rarely preserved in the fossil record, it is difficult to determine which kind of tissue was located between the amphicoelous or platycoelous centra of extinct amniote clades. Romer (1956) suggested that in the amphicoelous centra of basal amniotes, there must have been modified notochordal material or fibrous tissue between the vertebrae.

### Zygapophyses

Not only the centra, neural spines, and ribs are relevant for the mobility of the vertebral column, but the zygapophyses are also important. Zygapophyses are processes arising from the neural arch, bearing intervertebral articulation facets that restrict vertebral rotation around the long axis. Zygapophyses are also called apophyseal joints, facet joints, and dorsal intervertebral joints. The anteriorly facing zygapophyses are called prezygapophyses, and those facing posteriorly are called postzygapophyses. The prezygapophyses have their articulation facet oriented dorsally, whereas the facets of the postzygapophyses are ventrally oriented. In extant reptiles and birds, the zygapophyseal surfaces are flat or only slightly curved. The mediolateral and anteroposterior angles of the zygapophyses differ between taxa and greatly influence the mobility of the vertebral column. Each of the two bony zygapophyses in the facet joint bears an articular surface that is covered by a usually thin layer of hyaline cartilage. There is a narrow joint cavity in between the articular surfaces, forming a synovial joint that allows translational movement only. The thickness of the cartilages and the joint cavity affects intervertebral mobility because the distance between the zygapophyses is not negligible.

The articular surface of the zygapophyses shows variation in relative size, both between taxa and along the vertebral column. If a vertebra has large and expanded zygapophyseal articular surfaces compared to the articular surfaces of the centrum, it has a higher ROM.

### Limitation in ROM by osteological stops and the osteological neutral pose (ONP)

Movement in the motion segment is limited most obviously by the osteological stops, i.e., the point where bones in a joint would collide. Osteological stops also can be observed well in fossils (Stevens, 2013). However, it has to be kept in mind that joints included cartilage and other soft tissues and that the vertebral column is also supported by muscles and ligaments. This means that the osteological stops constrain the maximal movement in the vertebral column and that the actual movement probably was less. Lateral movement is limited by osteological stops mostly formed by the centrum and the zygapophyses, but also by cervical ribs. Dorsal movement is mostly restricted by osteological stops formed by the zygapophyses and the neural spine and potentially the dorsal margin of the centrum, whereas ventral movement is restricted by osteological stops formed by the ventral margin of the centrum and also by the zygapophyses. It thus becomes apparent that the zygapophyses play a crucial role in the mobility of the vertebral column.

Three directions of movement are commonly recognized in the vertebral column: lateral, ventral, and dorsal, reflecting two of the three degrees of freedom. Note that in medicine and in some biological publications, ventral movement is termed ‘flexion’ and dorsal movement is termed ‘extension’.

The habitual posture of the neck in a terrestrial amniote commonly coincides with the osteological neutral pose (ONP) (Stevens, 2013). The ONP is defined as the specific pose of the vertebrae in articulation, where two adjacent vertebrae are held by the animal with 100% overlap of the zygapophyses (Taylor, Wedel & Naish, 2009). Whereas the ONP approach is not without problems, as noted by Dzemski & Christian (2007) and Nagesan, Henderson & Anderson (2018), it remains a viable approach because it makes the least assumptions.

### Inferences from morphology and biomechanics on the plesiosaur intervertebral joint

A prerequisite for building a finite element model of a motion segment is the formulation of hypotheses about the nature of the soft tissue intervening between the two vertebral bodies and their spatial arrangement. Two aspects are of primary importance: the spacing of the vertebra and the types of intervertebral joints. Let us first consider the spacing. Plesiosaurs are commonly found as articulated or partially articulated skeletons due to their pelagic habits which favor preservation in conservation deposits. Citing only a few examples, such as the Hettangian and Sinemurian deposits of southern England (Benson, Evans & Druckenmiller, 2012; Benson, Evans & Taylor, 2015), the Posidonienschiefer Formation of Germany (O’Keefe, 2004), the Cretaceous dark mudstones and chalks deposited in the Western Interior Seaway of North America (e.g., O’Keefe & Chiappe, 2011; Schumacher & Martin, 2016), and the inland Eromanga Sea of Australia (Zammit, Daniels & Kear, 2008), these fossils provide good evidence for the natural position of the vertebra. Even the oldest and only Triassic plesiosaur skeleton, from the Rhaetian Exter Formation of Germany (Wintrich et al., 2017), preserves such evidence. This skeleton as well as many others cited above preserve necks in tight articulation, showing an intervertebral spacing of 1 to 2 mm (e.g., Wintrich et al., 2017; Fig. 2C; Benson, Evans & Taylor, 2015; Fig. 13; Andrews, 1910) and very tight articulations of the zygapophyses. This preservational evidence is consistent with anatomical evidence of zygapophyseal overlap in the ONP discussed above, which also indicates short distances between the centra. An apparent exception is the leptoclidid plesiosaur *Nichollssaura borealis* that preserves considerably wider intervertebral spacing of 7 to 16 millimeters (Nagesan, 2017) but differs from *Cryptoclidus* and other small-headed plesiosaurs in its reduced number of only 24 cervical vertebrae (Druckenmiller & Russell, 2008; Druckenmiller & Russell, 2009).

In the case of plesiosaurs, conventional wisdom such as ’all reptiles have a synovial joint between adjacent centra’ (Hypothesis 1) is pitted against the possible presence of an IVD (Hypothesis 2). Let us first consider Hypothesis 1, a synovial joint developed on the platycoelous or slightly amphicoelous vertebral centra of plesiosaurs. The medial inclination of the zygapophyses in plesiosaur vertebrae in connection with a flat synovial joint between adjacent centra would reduce the mobility to a minimum. As a derived cryptoclidid plesiosaur, *Cryptoclidus eurymerus* reached nearly the maximum of zygapophyseal inclination, which is 82° as measured in IPB R324 (but also illustrated by Andrews, 1910; Figs. 79 and 80). The flat synovial join does not allow rotation about the joint long axis, but rather a translation along the joint surface. Furthermore, the translation along the surface would be restricted by the zygapophyses, which would mean that the plesiosaur neck has no mobility at all.

Although crocodiles have a synovial joint between their vertebrae (Wettstein, 1962), they differ in having procoelous centra, and thus the synovial joint functions as a mobile ball-and-socket joint. This shape allows three degrees of freedom about the axis, which increases mobility. Thus, accepting the first hypothesis of a flat synovial joint connecting platycoelus centra would result in the minimal ROM for plesiosaurs.

Under hypothesis 2, there would be a similar structure to that seen in mammals, an IVD, in between the centra in the plesiosaur neck. This would increase mobility to the point where other features become limiting, such as the zygapophyses. An IVD would provide the maximal ROM apart from a ball-and-socket joint. A cartilaginous structure of the kind seen in bird dorsal intervertebral joints, consisting only of hyaline cartilage and fibrocartilage but lacking a nucleus pulposus, appears implausible because it also would lead to a minimal ROM. We thus conclude that the most likely situation in plesiosaurs is an IVD.

## Material and Methods

### Material

The Middle Jurassic plesiosaur we studied is a mounted skeleton (IPB R324) of the cryptoclidid plesiosaur *Cryptoclidus eurymerus. C. eurymerus* is a derived plesiosaur which exhibits some basal plesiosaur characters which makes the taxon useful for determining mobility in plesiosaur necks. The skeleton is from the Lower Oxford Clay (Callovian) of Peterborough, England, and is on display at the Goldfuß Museum of the Institute of Geosciences, University of Bonn, Germany. IPB R324 was purchased from the foremost collector of Oxford Clay fossils, Alfred Leeds, through the Bonn fossil dealer Bernhard Stürz in 1911. Original Leeds collection stickers on the bones suggest that the skeleton is a composite, consisting of three individuals (written comm., Jeff Liston, 2015). The neck, skull and rest of the axial skeleton, however, derive from a single individual (written comm., Jeff Liston, 2015). Another individual is represented by three of the four flippers, with the right hind flipper pertaining to a third individual. The cervical vertebral column of IPB R324 consists of 32 vertebrae which are mounted in correct anatomical order, followed by three pectorals. However, four of the cervical vertebrae turn out to be entirely casts which had been inserted in different places to fill size gaps during mounting, leaving 28 original vertebrae. This insertion during mounting presumably also was motivated by the report of “about 32 cervical vertebrae for *Cryptocleidus*” by Andrews (1910) p. 164. Andrews (1910) did not explain the meaning of “about”, i.e., whether there is intraspecific variation or uncertainty because of incomplete preservation (see also Brown, 1981). However, in this study, we will use the count of 32 cervical vertebrae. It has been suggested that the neck is more mobile in its anterior third (as reviewed by Noè, Taylor & Gómez-Pérez, 2017; see also Nagesan, Henderson & Anderson, 2018) and less mobile at the base. To take these variations into account, we took a well preserved posterior middle cervical for our model. Cervical vertebrae 21 to 23 were removed from the IPB mount for µCt scanning, and all cervical vertebrae were removed for morphological study. Vertebra number 22 is the best preserved one in this region, showing little deformation and only slight restoration at the tip of the neural spine. µCt scanning revealed that cervical 21 and 23 are too deformed and too restored to serve in the finite element analysis.

### Methods

#### Micro-computed tomography scanning and 3D reconstruction

Surface models based on micro-computed tomography (µCt) scans are the basis for the quantitative and qualitative description of vertebral morphology and features relevant for neck mobility in particular. The µCt scans were recorded with a v—tome—x s scanner manufactured by GE phoenix—X-ray (Wunstorf, Germany) and operated by the Division of Paleontology. A total of 1,000 images with an exposure time of 667 ms and an average factor of four were recorded per scan. Voxel size was 190.97 µm, the voltage was set at 120 mV, and the current was set to 100 µA. The image stack consisting of 600 images generated by the software VG Studio Max from the rotational X-ray images collected was transformed from .jpg files to DICOM files for 3D model building.

A three-dimensional surface model of cervical vertebra no. 22 was generated from the DICOM files using Avizo 8.0.1 (FEI, Hillsboro, Oregon, USA). The surface model was then duplicated to build a single motion segment. Duplication was necessary because a motion segment could not be built from the scans of two adjacent vertebrae because of poor preservation (see above). For an anatomically accurate positioning of the two vertebral models (cervical vertebra no. 22 and its duplicate) relative to each other, the spinal canal as well as the articular surfaces of the zygapophyses and the centra was used. The anatomical positioning of the two vertebrae was also conducted in Avizo.

#### Finite element modeling

##### General anatomical assumptions for the model.

We employ models that are from a biomedical background and were developed for mammalian intervertebral joints. As noted above, the models were easy to adapt for this study because of the inferred presence of an IVD in plesiosaurs. Therefore, we modeled the plesiosaur motion segment including a very simplistic IVD between the two vertebral centra. The IVD was represented in the model by an annulus ring and a nucleus pulposus. The implemented nucleus was based on the nucleus pulposus of a human cervical IVD. According to a series of studies (e.g., Ecklin, 1960; Oda, Tanaka & Tsuzuki, 1988; Töndury & Theiler, 1990; Tonetti et al., 2005), the nucleus pulposus of the human cervical IVD mainly consists of fibrocartilaginous tissue. Therefore we did not implement an incompressible fluid which is most often used for models of the lumbar IVDs of humans.

##### Model dimensions and material properties.

**Figure 1:**
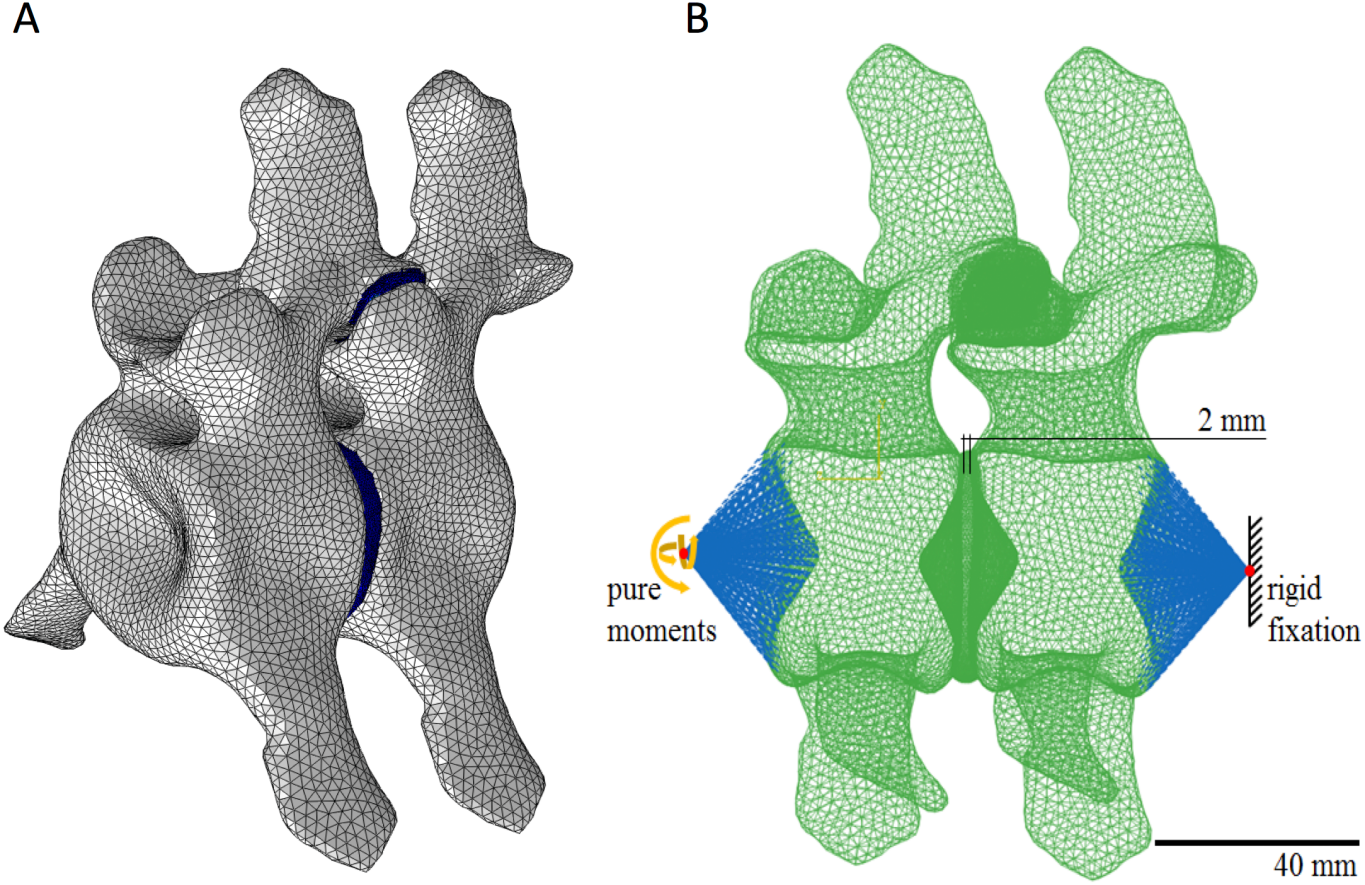
Finite element model with a dorsal intervertebral disk length of 2 mm. Finite Element (FE) model of two middle to posterior cervical vertebrae and the inferred intervening soft tissue articulation of *Cryptoclidus eurymerus* (IPB R 324). The FE model employs an IVD length of 2 mm at the dorsal margin of the IVD and consists of 68,212 elements and 323,480 nodes. Models with 1 mm and 3 mm dorsal IVD lengths were also used, see text for details. The models were generated from a CT scan of vertebra 22 in the software Polyworks. (A) oblique left lateral view of the 2-mm model. (B) midline saggital section of the 2-mm model. Color conventions: Gray is the bone material. Dark blue is the soft tissue between the two vertebrae. Green meshwork is the bone material including the bony endplate, solid green is the nucleus matrix, blue lines represents the applied forces. For material properties, see Table 1.

There is no data available concerning the original length of plesiosaur IVDs. However, based on the observed close spacing of the cervical vertebral centra in most plesiosaur fossils and the ONP derived from vertebral morphology (see above), three different models were built, only differing in IVD length (Fig. 1). The length of the IVD at its dorsal margin in the three different models was approximately 1, 2, and 3 mm to model IVDs of that minimal length. Furthermore, articular cartilage with a thickness of approximately 0.2 mm was modeled within the gaps between the articulated pre- and postzygapophyses of the left and the right side of the vertebrae, simulating the two facet joints (Fig. 1). Although a thickness of 0.2 mm for the articular cartilage might appear unrealistically thin, the virtual articulation of the vertebra in the model that gave the best fit, and overlap of the zygapophyses resulted in a small gap of 0.2 mm. Wider gaps could only be created with poorer fits of the zygapophyses.

The zygapophyseal articular cartilage also included contact elements, which were needed to simulate the mobility constraints generated by the facet joints. We neglected the presence of a facet joint capsule in order to obtain the widest ROM possible allowed by the morphology of the vertebrae, i.e., by the osteological stops. For this purpose, we also decreased the stiffness value (Young’s modulus) of each soft tissue component to the minimum required for a stable finite element simulation (Table 1), thus maximizing possible ROM. In contrast to the FE models of the human IVD, we did not include any fiber layers within the annulus ring due to the lack of data for plesiosaurs. Furthermore, we implemented the bone tissue of the vertebrae using rigid material behavior in order to avoid any contribution to the overall ROM by the deformation of the vertebral bodies.

##### Model building, FE analysis, and total ROM calculation.

The FE model generation, including all soft tissue components, material settings, and boundary conditions, was performed using ANSYS APDL 15.0 (Ansys Inc., Canonsburg, Pennsylvania, United States). The final models consisted of 68,212 to 87,220 elements and 323,480 to 427,569 nodes, depending on the setting for the IVD length (Fig. 1). The shorter the distance the between the vertebrae, the more nodes were required, because a more dense mesh of nodes and elements was required for modeling accuracy.

**Table 1:** Material properties used in the FE simulations of the plesiosaur neck motion segment. E = Young’s modulus.

**Tissue**	**Material setting**
Anulus matrix	E (MPa): 0.8, *υ* (Poisson ratio): 0.45
Nucleus matrix	E (MPa): 0.5, *υ* (Poisson ratio): 0.45
Bone tissue of vertebrae	Rigid
Bony endplates	E (MPa): 600.0, *υ* (Poisson ratio): 0.3
Facet cartilage	E (MPa): 100.0, *υ* (Poisson ratio): 0.3

The posterior surface of the posterior vertebra was fixed concerning all six degrees of freedom (Fig. 1). Regarding the FE simulations, we applied pure bending moments to the anterior surface of the anterior vertebral body using a coupling node. The bending moments included flexion, extension, lateral bending and axial rotation. We are aware that this is an oversimplification of the real situation where, in the living animal, muscles inserting on different regions of the neural arch would have exerted these moments which then would have been transmitted to the vertebral centrum via the peduncles of the neural arch and for the ventral muscles from the cervical ribs.

**Figure 2:**
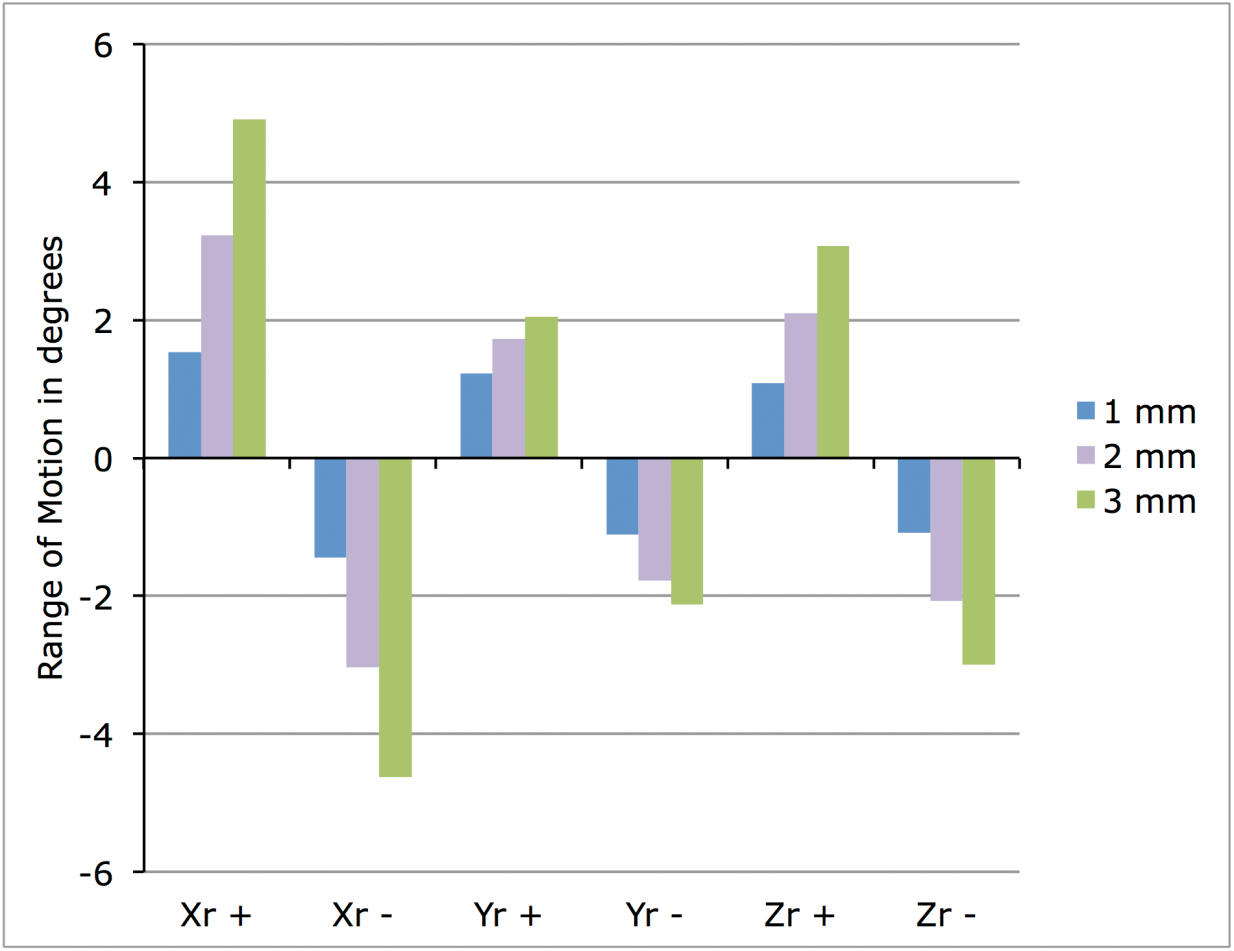
Graph of ROM in the modeled motion segment depending on the length of the IVD. Obviously, the longer the IVD, the greater the ROM in fractions of degrees. Xr +, flexion (dorsal); Xr −, extension (ventral); Yr +, left lateral deflection; Yr −, right lateral deflection; Zr +, clockwise rotation around long axis; Zr −, anticlockwise rotation around long axis (from the perspective of the animal).

In addition to the material parameters and the intervertebral space, we also had to assume the value for these moments since obviously no data are available for the plesiosaur. We gradually increased the applied bending moment until the finite element simulation became unstable, which was above 4 Nm. We applied the same bending moment in all load directions, as is customary in finite element simulations of the human spine. Each moment was applied separately. Thus, a total of six load cases in positive and negative direction of flexion-extension, lateral bending, and axial rotation were computed for each of the three models (intervertebral spacing 1, 2, and 3 mm), resulting in a total of 18 simulations (Fig. 2).

The final FE simulations were conducted using Abaqus 6.11 (Simulia, Dassault Systèmes, Vélizy-Villacoublay, France). The ROM for each load case was measured at the anterior coupling node, which was also used for load application (Fig. 1). To compute total neck mobility of the IPB *Cryptoclidus* specimen, we multiplied the ROM obtained for each load case by the number of intervertebral joints in the neck. Based on the total of 32 cervicals, the fused atlas-axis joint, and the inclusion of the joint between the last cervical and the first pectoral, we employ a count of 31 joints. We are aware that this is an oversimplification, but it makes the least assumptions based on our data.

## Results

### ROM in the modeled motion segment

The three different analyses were based on the different intervertebral spacing and the different load cases (Fig. 2). The ROM increased with increased IVD length in all load cases, but it is generally small, ranging from at most 5° to less than one degree. In the load cases with the 3-mm IVD, dorsal and ventral ROM is above 4°, decreasing to about 3° in the 2-mm IVD model. At an IVD thickness of 1 mm, about 1° is obtained. Lateral ROM is about 2° in the 3-mm IVD, 1° in the 2-mm IVD, and also 1° in the 1-mm IVD (Fig. 2). Rotational ROM is about 3° in the 3-mm IVD, about 2° in the 2-mm IVD, and also about 1° in the 1-mm IVD (Fig. 2).

The difference between load cases is least for an IVD thickness of 1 mm, where ROM never exceeds 1°. With increasing IVD thickness, differences between load cases become more apparent, and the greatest ROM is seen in the dorsal and ventral flexion at 3 mm IVD thickness, while rotational ROM is least (Fig. 2). Interestingly, relative ventral and dorsal ROM is similar in all analyses, with dorsal ROM always being slightly greater than ventral ROM. Left lateral and right lateral ROM as well as left and right rotational ROM are closely similar.

### Total ROM in the *Cryptoclidus* neck

**Figure 3:**
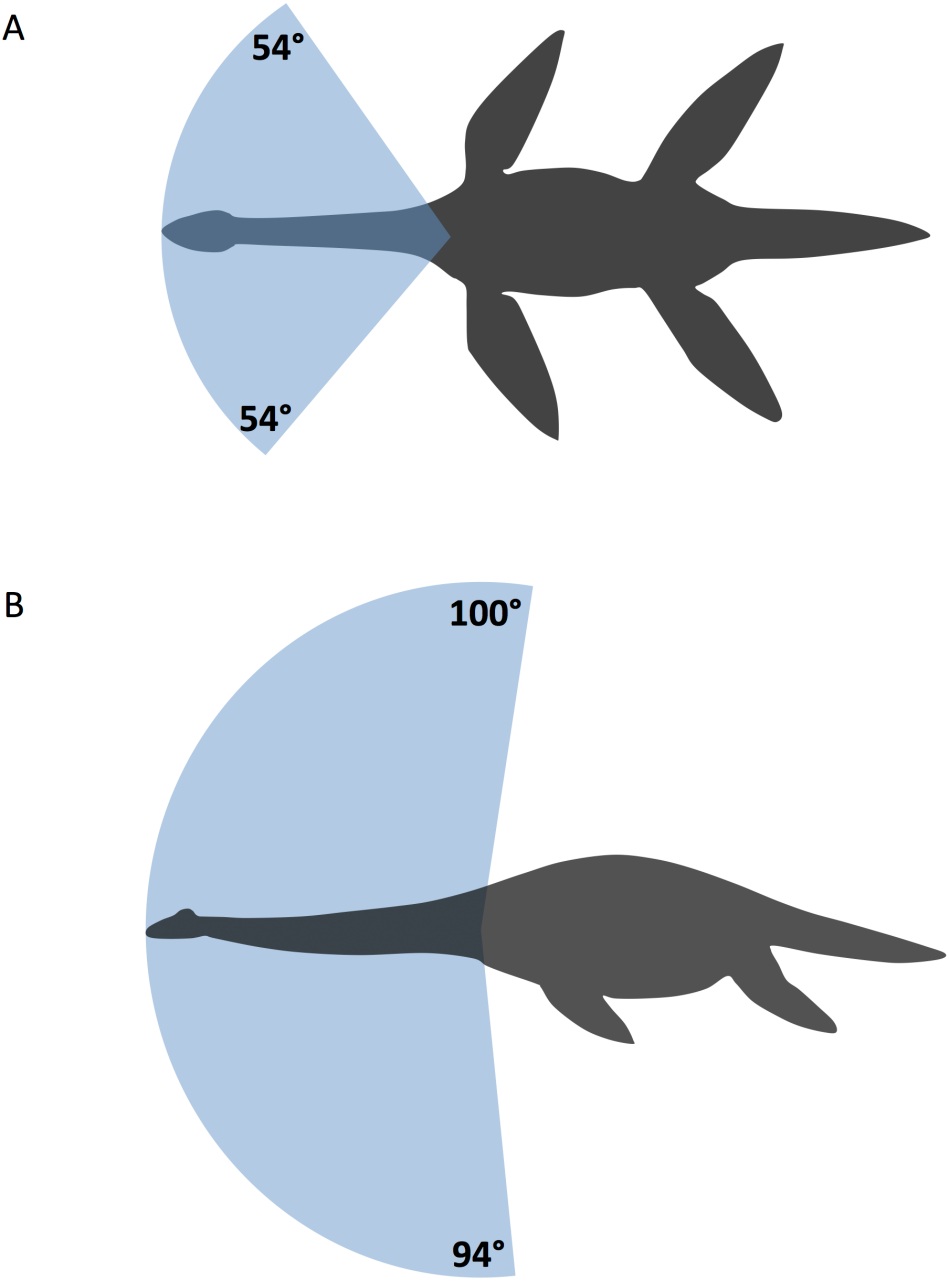
Diagram of maximal neck movement in *Cryptoclidus eurymerus* with an intervertebral disc length of 2 mm. (A) movement in lateral direction projected onto a dorsal view of generalized plesiosaur silhouettes and (B) movement in dorsoventral direction projected onto a lateral view of a generalized plesiosaur silhouettes. The fans indicate the maximum ROM (Table 2, see also ‘Discussion’ section), rounded to the nearest full degree, summed up from the movement in the modeled motion segment. This is based on the multiplication by the number of 31 functional intervertebral joints, assuming that there were 32 cervical vertebrae and that the atlas and axis were fused, as described by Andrews (1910) and confirmed in IPB R 324.

Of course, total ROM of the *Cryptoclidus* neck depends crucially on IVD length. Based on the 31 intervertebral joints in the neck of the Bonn specimen of *Cryptoclidus eurymerus* IPB R324, we obtained three values for each direction (dorsal, ventral, and lateral) of total neck mobility. Assuming an IVD length of 1 mm, we obtain total ROM of only 47° for dorsal flexion, 45° for ventral flexion, and 36° for lateral flexion (Fig. 3; Table 2). Assuming an IVD length of 2 mm, we obtain total ROM of 100° for dorsal flexion, 94° for ventral flexion, and 54° for lateral flexion. Finally, for the greatest likely IVD length of 3 mm, we obtain a total ROM of 152° for dorsal flexion, 143° for ventral flexion, and 65° for lateral flexion. Thus, the greatest mobility was clearly present in the dorsal direction, closely followed by the ventral direction. Lateral flexion was most limited in *Cryptoclidus*, never even approaching 90°. At an intermediate IVD length of 2 mm, *Cryptoclidus* would have been able to bend its neck slightly beyond a 90° angle in dorsal and ventral direction, and only with an IVD length significantly greater than 3 mm, the neck could have been bent into a full half circle in dorsal or ventral direction and a quarter circle in lateral direction (Fig. 3). Taking *Cryptoclidus* as representative, mobility in the neck of long-necked plesiosaurs was rather limited, in strong contrast to the high number of cervical vertebrae intuitively suggesting great, snake-like mobility. Rotational ROM is in general of limited importance for the total ROM because geometrically the rotation around the neck long axis cannot be translated into increased lateral or dorsoventral deflection but actually is part of it as soon as the neck is moved outside a strictly horizontal or vertical direction. This relationship has been investigated in detail in the human spine (e.g., Yoganandan et al., 1996; Kapandji, 2019) and applies to plesiosaurs as well.

## Discussion

### Methodological issues

Accurate FE analyses require a sufficient approximation of the reality. Most importantly, this includes the geometry of the object, its material properties, and the prevalent boundary conditions. Modeling and simulating biological tissue using finite element has become very popular in research and product development (Shirazi-Adl, Ahmed & Shrivastava, 1986; Goel et al., 1988; Yoganandan et al., 1996). However, these studies remain challenging due to the complexity and inhomogeneity of biological materials compared to technical ones (Freutel et al., 2014; Viceconti et al., 2005; Schlager et al., 2018). The material parameters used in this study are not based on experimental data because we are dealing with fossils. Instead, we use well-justified assumptions. Due to the lack of knowledge about the mechanical properties of soft tissues of plesiosaurs in general, and *Cryptoclidus eurymerus* in particular, we reduced the stiffness of the modelled IVD to a minimum, below those values that have been used in models of extant IVDs.

**Table 2:** Calculated total range of motion in degrees for dorsal flexion, ventral flexion, and lateral flexion in dependence of IVD length in the *Cryptoclidus eurymerus* neck based on specimen IPB R 324. Total ROM was obtained by multiplying the ROM for the modeled motion segment in dorsal, ventral, and lateral direction with the number of 31 functional intervertebral joints (assuming that there were 32 cervical vertebrae and that the atlas and axis were fused, Andrews, 1910).

**IVD length**	**Dorsal ROM in MS**	**Total ROM dorsal**	**Ventral ROM in MS**	**Total ROM ventral**	**Lateral ROM in MS**	**Total ROM lateral (mean of L/R)**
1 mm	1.53	47	1.44	45	1.16	35.8
2 mm	3.22	100	3.0	94	1.75	54.3
3 mm	4.9	152	4.6	143	2.09	64.9

**Notes.**

MS motion segment L left R right ROM range of motion in degrees

By using this material setting, we were able to estimate the greatest ROM that can be achieved with the given vertebral anatomy. Assuming a greater stiffness of the modeled plesiosaur IVD would only result in an even less mobile neck, supporting our overall conclusion of low neck mobility. Another limitation of our analysis concerns the inferences and missing data about the true IVD thickness and its material properties. Therefore we modeled and tested three different IVD lengths based on the range of observed intervertebral spacing in fossil specimens. We only worked with one cartilage thickness in the facet joints because any significant deviation from this thickness resulted in unrealistically disarticulated joints. However, further investigations concerning the true intervertebral spacing and the thickness of the soft tissue in the facet joints in plesiosaurs are clearly needed in order to obtain more reliable estimations about the total ROM.

Our study is based on the model of a single vertebra obtained from µCT data. The vertebra was used to create a single motion segment by simply duplicating it. This approach is justified because of the very gradual morphological change along the cervical vertebral column (e.g., Andrews, 1910; Brown, 1981, and personal observations on IPB R324) which in turn is related to the high number of segments. The error caused by duplicating the vertebra is assumed to be relatively small compared to the error resulting from the missing soft tissue data. We note that our surface model is based on a fossil specimen, not on a model of the vertebrae. This is also the explanation for the slight difference between the left and right lateral bending values, which theoretically should be the same. The difference is probably due to the slight asymmetries in the scanned vertebra.

The resolution of the model meshes (node density) was high. Using an even denser mesh would not have offered further improvement in the accuracy of the models since the error introduced by the lack of knowledge of actual tissue parameters is assumed to be much higher than that introduced by lower mesh density.

Finally, we offer justification of our approach of using a method from human biomechanics for understanding a Mesozoic marine reptile. The strength of this approach is that it is a mature and well established method from a large and applied field of research and thus will produce reliable and reproducible results. The weakness lies in the assumption about soft tissue input parameters that cannot be measured in fossils.

### Reconstruction of the ROM in the *Cryptoclidus* neck

In pistosauroid evolution, three major steps in neck evolution can be recognized, from pistosaurids (non-plesiosaurian pistosauroids) to basal plesiosaurs (outside of Cryptoclidia sensu Benson & Druckenmiller, 2014) to derived plesiosaurs, i.e., Cryptoclidia.

In pistosaurids such as *Pistosaurus*, *Augustasaurus*, and *Yunguisaurus*, orientation of the prezygapophyses is nearly horizontal (Rieppel, Sander & Storrs, 2002), as in all other stem eosauropterygians, and the articular surface area is large in comparison to the vertebral centrum. In pistosaurids excluding *Bobosaurus forojuliensis*, the number of cervical vertebrae is between 37 and 49 (Soul & Benson, 2017). In basal plesiosaurs, the numbers of cervical vertebrae decreases slightly in comparison to pistosaurs but increases in the derived plesiosaurs again (Soul & Benson, 2017). Pliosauromorphs may greatly reduce the number of cervicals, of course. The zygapophyses in basal plesiosaurs are medially inclined as opposed to non-plesiosaurian pistosauroids (see coding of character 169 in Benson & Druckenmiller, 2014). In basal plesiosaurians, zygapophyses are inclined at about 45°, e.g., in *Rhaeticosauru* s (Wintrich et al., 2017) and *Stratesaurus* (Benson, Evans & Taylor, 2015) and in derived plesiosaurs, they may be inclined at over 80°, which is seen in *Cryptoclidus eurymerus* (Andrews, 1910). It can be hypothesized that this would restrict lateral mobility over dorsoventral mobility, as shown by our results and previous studies, such as Noè, Taylor & Gómez-Pérez (2017).

To obtain total neck mobility, we multiplied the mobility modeled for a specific cervical motion segment by the number of vertebral joints (31), excluding the fused atlas-axis joint. However, note that this is an oversimplification because the actual ROM was less because the neck was not moved in a single joint but in each intervertebral joint and the full mobility was only achieved in the cranialmost segment. Our calculation also is only a first approximation since it appears likely that the deflection degree of the motion segment differs between the anterior and posterior parts of the cervical column, as for example is seen in elasmosaurs (e.g., Zammit, Daniels & Kear, 2008; Otero, Soto-Acuña & O’Keefe, 2018). We are thus aware that the most accurate result for total neck mobility would be obtained if we were to model the entire cervical column and add up the maximum ROM for each motion segment. The next step would be to characterize the distribution of mobility along the neck from anterior to posterior, which would be particularly interesting in the extremely long-necked elasmosaurs; similar studies have been conducted for sauropod dinosaurs (e.g., Dzemski & Christian, 2007; Christian & Dzemski, 2011). However, as noted in these studies and others (Andrews, 1910; Noè, Taylor & Gómez-Pérez, 2017) and confirmed by personal observations, cervical vertebral morphology does not vary sufficiently along the neck to radically alter our conclusion that the plesiosaur neck was relatively immobile. Finally, there is the question of the mobility at the base of the neck, possibly provided by highly mobile joints in the pectorals. Such joints have not been described in the literature to our knowledge, and we note that high mobility at the base of the neck would not solve the paradox of the immobile but long neck itself. A neck that is mobile at its base but stiff throughout only seems marginally better suited for prey acquisition than a completely stiff neck.

Our reconstructions, based on the medical biomechanical investigation of neck mobility, are consistent with the inferences on mobility based on osteological stops and zygapophyseal inclination in the neck vertebral column (discussed above), suggesting that in *Cryptoclidus* dorsoventral mobility was greater than lateral mobility because of the strongly medially inclined zygapophyses. The results are also consistent with inferences based on morphological characters in phylogenetic data matrices (e.g., Benson & Druckenmiller, 2014).

However, our results do not agree with the qualitative anatomy-based interpretations by Noè, Taylor & Gómez-Pérez (2017) favoring ventral mobility of the neck. On the other hand, the results obtained by 2D analog modeling by Zammit, Daniels & Kear (2008) are more consistent with ours, giving a similar range of values of ROM per motion segment. Because of the higher number of vertebrae in elasmosaurs, Zammit, Daniels & Kear (2008) obtained considerably greater total neck mobility than we did in *Cryptoclidus*, especially in the 1- and 2-mm IVD models*.* Zammit, Daniels & Kear (2008) differ in finding roughly equal mobility in all directions (75–177°), unlike the clearly lower lateral mobility we obtained (<67°). Finally, the most recent study by Nagesan, Henderson & Anderson (2018) also found high mobility in a relatively long-necked plesiosaurs, the leptoclidid *Nichollssaura*, but this taxon appears to differ from other small-headed plesiosaurs in the number of its cervical vertebrae having been reduced to only 24. To compensate for this reduction but maintaining neck length, the lineage may have evolved wider intervertebral spacing, and we doubt whether this taxon is representative for long-necked and short-headed plesiosaurs in general.

Our investigation is an approximation, but it shows clearly which morphological features and morphometric variables determine mobility of the mid to posterior region of the neck. Although several characters in addition to IVD length potentially influence neck mobility in our model, the most important of these is zygapophysis morphology. Specifically, anteroposterior length of the articular surface, thickness of the joint cartilages between the pre- and postzygapophysis, and medial inclination are characters that need to be understood and measured to determine motion segment mobility and thus that of the entire neck.

### Influence of reconstruction of IVD

The reconstruction of the IVD obviously also influences the models. As already noted by Romer (1956), the question of what kind of soft tissues intervene between the two bony centra in a motion segment remains open. Romer speculated that the “conjoined hollows may be filled with modified notochordal material or fibrous tissue” (Romer, 1956, p 223). We argue that the intervertebral space in the plesiosaur motion segment was not developed as a synovial joint because a synovial joint would only have allowed translational movement because of the amphicoelous or platycoelous centrum shape. An intervertebral space purely consisting of a disc of fibrocartilage would movable either because of the very limited elasticity of this tissue both under tension and compression. Thus, we arrived at the conclusion that plesiosaurs possessed an IVD of some kind. In the IVD, there would have been at least two kinds of tissues, the distribution of which needed to be considered for our model. These tissues are the cartilage of the annulus fibrosus and the notochord-derived tissue of the nucleus pulposus. While the material parameters were estimated as discussed above, we also need to consider the anatomical arrangement of the different tissue types. We reconstructed the annulus fibrosus on the convex part of the articular surface outside of the sunken area. The remainder of the intervertebral spaced was filled up by the nucleus pulposus. The question of the exact nature and distribution of the tissues filling the intervertebral space is beyond the scope of this paper.

### Influence of muscles and ligaments

When considering mobility, the influence of muscles and ligaments has to be discussed as well as other features, both from the perspective of functional morphology in living animals (e.g., Dzemski & Christian, 2007; Taylor, Wedel & Naish, 2009; Stevens, 2013; Taylor & Wedel, 2013) and from the perspective of the fossil record, i.e., the posture of the neck in articulated fossils. In taphonomic studies (e.g., Reisdorf & Wuttke, 2012), it has been shown that neck mobility changes in different taphonomic stages: (1) First, in the living animal, mobility is at a minimum. Here, all muscles and ligaments remain connected to the cervical vertebral column and furthermore all nerves are intact, restricting any mobility beyond the osteological stops. (2) Second, mobility is greater when the animal is freshly dead. Despite all muscles and ligament still being intact, in a dead animal, an experimental hyperextension is unproblematic, leading to greater mobility than in the living animal (Christian & Dzemski, 2011). (3) Third, mobility is maximal when all muscles and ligaments are removed from the skeleton by decay processes. Here, only the osteological stops, basically provided by the zygapophyses, the neural spine, the centrum, and the ribs, determine mobility. This situation of decay applies to articulated skeletons in marine deposits, such as those of plesiosaurs from the Lower Jurassic of Lyme Regis (UK) and the Posidonienschiefer Formation of Holzmaden (Germany) and the equivalent Toarcian rocks of Whitby (UK). The articulated plesiosaur specimens preserved *in situ* from these deposits general show a straight neck (e.g., Beardmore et al., 2012; Benson, Evans & Druckenmiller, 2012), corroborating the limited mobility of the neck in long-necked plesiosaurs as noted by previous authors (Watson, 1924; Watson, 1951; Cruickshank & Fordyce, 2002; Zammit, Daniels & Kear, 2008; Noè, Taylor & Gómez-Pérez, 2017).

### Functional and paleobiological implications

We show that the neck of the plesiosaur *Cryptoclidus eurymerus* has a limited ROM in dorsal bending (extension), ventral bending (flexion), and especially lateral bending. The term ’limited’ refers to a maximal total neck mobility in dorsal and ventral direction combined of less than 160° and in lateral direction towards one side of less than roughly 70° (Table 2), far less than the snake-like necks hypothesized in earlier studies. Especially given that these values are upper limits of mobility, mobility was also significantly less than what has been found in previous quantitative studies (Zammit, Daniels & Kear, 2008; Nagesan, Henderson & Anderson, 2018). We suspect that most plesiosaurs had a limited ROM in the neck, inconsistent with their great relative and absolute neck length that intuitively would be associated with great flexibility. The long necks clearly evolved in basal eosauropterygians, retaining the lateral mobility ancestral for amniotes as indicated by the horizontal zygapophyses that are wider than the centra (Rieppel, 2000; Sato et al., 2014). While lateral undulation appears to have become less important in swimming in taxa such as *Yunguisaurus* (Sato et al., 2014), the laterally mobile neck was retained. This raises the question as to why at least some plesiosaurs retained these long necks but reduced mobility, and how reduced mobility fits into the evolution of the long-necked plesiosaur body plan.

After the swimming style in the lineage leading to plesiosaurs evolved from axial undulation to paraxial underwater flight at some time in the Late Triassic (Benson, Evans & Druckenmiller, 2012; Sato et al., 2014; Wintrich et al., 2017), the long neck was no longer needed in undulatory locomotion if, indeed, the neck was ever used in undulatory locomotion. However, the long neck would have had more functions than use in locomotion. Long necks evolved in several amniote clades (birds, sauropod dinosaurs, tanystropheids, and eosauropterygians) for improving food acquisition by extending the reach and maneuverability of the head as the organ for food uptake, both on land and in the sea (e.g., Preuschoft et al., 2011; Stevens, 2013). For biomechanical reasons, a head borne by a long neck must be small, obviously limiting bite size. For piscivores such as plesiosaurs, this means that they must have fed on fish and squid not much larger than their head because the prey would have been swallowed whole. To obtain enough of these relatively small (compared to the mass of the animal) food items, a sufficient prey density would be required, especially if plesiosaurs had a high basal metabolic rate (Wintrich et al., 2017; Fleischle, Wintrich & Sander, 2018). Such high prey density is provided by schooling fish and squid.

A considerably variety of functional interpretations involving feeding have been proposed for the long plesiosaur neck, as reviewed by Noè, Taylor & Gómez-Pérez (2017). Alternatively to these, there is the hypothesis that in plesiosaurs, the long neck is best understood as a camouflage adaptation. This idea was first briefly advanced by Massare (1988) and Massare (1994) with regard to visual camouflage, but this hypothesis has not been given serious consideration since. However, the idea is worth exploring further, albeit in a speculative fashion, in the light of our inference of limited neck mobility. In addition to visual camouflage, hydrodynamic camouflage (Montgomery, Coombs & Halstead, 1995) needs to be considered as well. Importantly, vision and the lateral line organ of fish operate at different ranges but probably in concert (Montgomery, Coombs & Halstead, 1995). Whereas the lateral line organ can only detect disturbances in the range of the body length of the fish (e.g., Palmer, Deffenbaugh & Mensinger, 2005), especially in front of a moving object, vision allows predator or prey detection at a range of tens of meters. Visual detection range depends on the size of the target, the contrast between target and background, lighting conditions, and water clarity.

We hypothesize that long-necked plesiosaurs hunting in schools of fish with a small head on a long neck would have increased the chance of catching the fish, i.e., predation success, because fish are sensitive to hydrodynamic disturbances through their lateral line organs (review in Montgomery, Coombs & Halstead, 1995). With the long neck and the small head, the hydrodynamic disturbance created by the approaching predator that could have been recognized by the prey, would have been much smaller than if the neck would have been shorter and closer to the bulk of the body. The bulk of the body may never have come close to the fish school given the length of the neck, the size of the individual fish, and the size of the school. The hydrodynamic disturbance of the plesiosaur head would possibly also have been similar to that created by a conspecific fish or a smaller predator, a hypothesis that is open to testing by computational fluid dynamics (CFD) modeling. In fact, such hydrodynamic camouflage has been described for the Triassic slender-bodied predatory fish *Saurichthys* (Kogan et al., 2015; Kogan, 2017) based on CFD. The slender body shape with rearward placement of the propulsive organ has evolved several times since in piscine predators, e.g., in *Lepisosteus* and *Belone* (Kogan et al., 2015; Kogan, 2017), presumably driven by selection for improved hydrodynamic camouflage.

In addition, as proposed by Massare (1988) and Massare (1994), there may have been a visual camouflage effect in that the long neck and small head would have hidden the true bulk of the predator. Distance vision in vertebrates depends on a variety of factors, as calculated by Nilsson, Warrant & Johnsen (2014) and MacIver et al. (2017). Ultimately, eye and pupil size, all else being equal, determine from which distance a target of specific size and contrast can be detected. Based on the Nilsson, Warrant & Johnsen (2014) model, a small fish with a pupil diameter of 3–5 mm would have able to see objects the size of a plesiosaur head at a distance of tens of meters in clear water during the day near the surface, questioning the importance of the visual camouflage effect in such conditions. Visual camouflage could have been important in low light conditions (greater depth, dawn, dusk), however, and in turbid coastal waters.

We now can speculate further about how a plesiosaur would have fed on a school of fish. Repeated fast attacks could have been an efficient way to harvest fish from the school without the school being able to detect the source of the attack, especially by lateral line organ. This mode of feeding would have been more efficient in less-clear shelf waters, where plesiosaurs might have enjoyed a competitive advantage over the large-eyed ichthyosaurs. This hypothesis is consistent with the types of sediment plesiosaur and ichthyosaur fossils are found in, reflecting habitat preference, i.e., more open-water in ichthyosaurs and more coastal in plesiosaurs.

The remaining issue is the evolutionary decrease in neck mobility from the laterally highly mobile neck in non-plesiosaurian pistosauroids to the limited mobility detected in this study. One hypothesis is that the mobile long neck originally played a role in lateral undulatory swimming, a function that was no longer relevant in plesiosaurs. While undulation of the neck during swimming has not been demonstrated in living animals (since there are no living aquatic animals with a long-necked pistosaur bauplan), a closer look at water snake locomotion might allow testing of this hypothesis. After the evolutionary reduction of neck mobility, with elongation of the neck mainly serving in hydrodynamic and visual camouflage, other functional constraints became important. Both during cruising and attack, the long neck needed to remain straight, and the long neck would have made steering difficult, with even small head and neck deflections having a great effect (Alexander, 1989, p. 137; Noè, Taylor & Gómez-Pérez, 2017; Troelsen et al., 2019). The straight posture in a mobile neck would require muscle effort to maintain (Troelsen et al., 2019). The evolution of osteological stops and thin intervertebral joints might have been energetically more advantageous than using muscle power to keep the neck straight, as indeed would fewer, longer vertebrae.

An interesting pattern is observed in the evolution from basal plesiosaurs to more derived long-necked species, i.e., *Cryptoclidus*, which is in the focus of this study. As noted, medial zygapophysis inclination evolutionarily increased from 45° to over 80°, suggesting a decreased lateral and increased dorsoventral mobility. This is what is born out by our calculation of total neck mobility, with dorsal and ventral flexion distinctly greater than lateral flexion, especially in the simulations with the longer IVDs. This raises the question of the adaptive background of this evolutionary change. Possibly, the pattern is linked to a hypothetical increase in swimming performance from early to later plesiosaurs. Increased swimming performance might have increased dorsoventral moments in the body (because of the dorsoventral movement of the flippers) which then would be transmitted to the neck (see also Alexander, 1989). Increased dorsoventral mobility might have served in dampening of such moments. However, while testable, this hypothesis would require extensive biomechanical modeling of a swimming *Cryptoclidus*, and we only point out the pattern here.

## Conclusions

One of the most distinctive features of plesiosaurs is their sometimes exceedingly long neck. Neck length, however, is convergently reduced in some large-headed forms in which the neck does not present a challenge. A classical plesiosaur taxon is *Cryptoclidus* from the late Middle Jurassic Lower Oxford Clay of the UK. We investigated the mobility of its neck using finite element analysis. First, we built a simple finite element model of a motion segment based on a complete and undistorted fossil vertebra from the posterior part of the middle cervical region. We then simulated the ROM of this segment using three different hypothetical IVD lengths (1 mm, 2 mm, and 3 mm). Finally, we calculated total neck ROM by multiplying segment ROM by the number of intervertebral joints in the neck (31) and find that neck mobility is surprisingly low in all directions. Assuming the greatest reasonable intervertebral spacing of 3 mm, we find a maximum lateral deflection in the neck of 67°, ventral deflection of 148°, and maximum dorsal deflection of 157°. We are aware that mobility probably varied along the neck and consider our results a first approximation open to further testing by, e.g., simulating an entire neck.

The limited mobility of the *Cryptoclidus* neck, especially sideways, is counter-intuitive given the serpent-like appearance of its neck and that of long-necked plesiosaurs in general. Limited mobility is, however, consistent with qualitative anatomical observations on cervical vertebral anatomy. We hypothesize that the reduced mobility of the neck in long-necked plesiosaurs compared to that of their ancestors is due to the function of the neck in hydrodynamic and visual camouflage combined with the high moments exerted on this neck during feeding and locomotion. If representative of other plesiosaurs, the greater mobility of the neck of *Cryptoclidus* in dorsoventral direction compared to the lateral direction may also be linked to the evolutionary change in locomotion from lateral undulation to underwater flight in ancestral plesiosaurs and the subsequent elaboration of this swimming style within the clade.

##  Supplemental Information

10.7717/peerj.7658/supp-1 Supplemental Information 1 3D model Cryptoclidus

## Additional Information and Declarations

### Data Availability

The following information was supplied regarding data availability: The specimen is accessioned to the paleontology collection of the Institute of Geosciences under the number IGPB R 324. The model is available at MorphoSource: https://www.morphosource.org/Detail/SpecimenDetail/Show/specimen_id/24882.
