# Supplementary material for: Neck mobility in the Jurassic plesiosaur Cryptoclidus eurymerus: finite element analysis as a new approach to understanding the cervical skeleton in fossil vertebrates
Source: PeerJ. 2019 Nov 6;7:e7658. doi: 10.7717/peerj.7658 (PMC6842296; doi:10.7717/peerj.7658)
Supplement: Supplemental Information 1 [file peerj-07-7658-s001.docx]

The 3D model of the *Cryptoclidus erymerus* is online in the morphosource database

Username: [tanja.wintrich@uni-bonn.de](mailto:tanja.wintrich@uni-bonn.de)

PW: passion
